# An appraisal of rehabilitation regimes used for improving functional outcome after total hip replacement surgery

**DOI:** 10.1186/1758-2555-4-5

**Published:** 2012-02-07

**Authors:** Tosan Okoro, Andrew B Lemmey, Peter Maddison, John G Andrew

**Affiliations:** 1 School of Medical Sciences (SMS)/School of Sport Health and Exercise Science (SSHES), Bangor University, Penrallt Road, Bangor LL57 2AS, UK; 2 School of Sport, Health and Exercise Science (SSHES), Bangor University, Holyhead Road, Bangor LL57 2PZ, UK; 3 Department of Orthopaedics, Ysbyty Gwynedd, Betsi Cadwaladr University Health Board, Penrhosgarnedd, Bangor LL57 2PW, UK

**Keywords:** Total hip replacement, Hip arthroplasty, Resistance training, Rehabilitation, Functional outcome, Muscle wasting

## Abstract

This study aimed to systematically review the literature with regards to studies of rehabilitation programmes that have tried to improve function after total hip replacement (THR) surgery. 15 randomised controlled trials were identified of which 11 were centre-based, 2 were home based and 2 were trials comparing home and centre based interventions. The use of a progressive resistance training (PRT) programme led to significant improvement in muscle strength and function if the intervention was carried out early (< 1 month following surgery) in a centre (6/11 centre-based studies used PRT), or late (> 1 month following surgery) in a home based setting (2/2 home based studies used PRT). In direct comparison, there was no difference in functional measures between home and centre based programmes (2 studies), with PRT not included in the regimes prescribed. A limitation of the majority of these intervention studies was the short period of follow up. Centre based program delivery is expensive as high costs are associated with supervision, facility provision, and transport of patients. Early interventions are important to counteract the deficit in muscle strength in the affected limb, as well as persistent atrophy that exists around the affected hip at 2 years post-operatively. Studies of early home-based regimes featuring PRT with long term follow up are needed to address the problems currently associated with rehabilitation following THR.

## Background

Symptomatic hip osteoarthritis occurs in 3% of the elderly [1] and is associated with poor general health status [2]. Treatment strategies for hip pain have usually involved conservative measures (analgesia, exercise, education, weight reduction), with surgical intervention (total hip replacement; THR) being the most effective treatment for end stage disease [3]. The National Joint Registry for England and Wales reports that the number of primary total hip replacements performed in England and Wales in 2009/2010 totaled 79413, which is a steady rise from the numbers reported in 2008/2009 (77608) and 2006/2007 (51981)[4].

The most common preoperative complaints by patients who elect to have THR are pain and loss of mobility [1,5]. It therefore follows that the most commonly reported outcomes of THR in the literature relate to pain relief and restoration of mobility [1]. It is clear that a major predictor of outcome after THR is the preoperative status - worse preoperative status is followed by a poorer absolute outcome as defined by several outcome measures [6]. Outcome studies of pain reduction and range of motion restoration, usually conducted 3 to 6 months after THR, indicate an overall satisfaction by patients and physicians [7]. However, outcome studies performed at least 1 year after THR reveal that limitations in physical function remain even in the absence of pain. These persisting impairments include decrements of 10-21% in muscle strength and postural stability of the involved hip relative to the non-operated hip at 1 year post-THR surgery [8,9], with these deficits still evident 2 years after surgery [2,10]. Interestingly, Long et al. [10] reported that muscle weakness during stance, along with deterioration of the Harris Hip score, were the most consistent findings in patients who developed loosening of the hip components; implying that muscle weakness may result in reduced protection of the prosthetic implant fixation.

Prior to surgery, there is a general deficit in muscular strength along the affected limb as compared to the contra-lateral (healthy) side in patients with unilateral hip osteoarthritis (OA) [11], and muscles such as the abductors, vastii, rectus femoris and psoas show marked atrophy. This is evidenced by reduced cross sectional area and an infiltration of non-contractile components; i.e. an average 10% increase in fatty infiltration of muscle (myosteatosis) in the affected limb compared to the healthy one [11]. As well as reducing muscle strength, myosteatosis also exacerbates fall risk [12]. This muscular dysfunction is likely to contribute to the reduced ambulatory capacity of OA patients, as loss of lower-limb muscle strength has been shown to predict the onset of activities of daily living dependence in the elderly [13]. Consistent with the persisting functional deficits following surgery, these atrophic changes about the hip are still evident up to 2 years following THR surgery [11]. Frail elderly persons with sarcopenia often undergo musculoskeletal-related surgery such as THR, and the hospitalisation-associated immobilisation further compromises the skeletal system, with potentially grave consequences [14]. Earlier operation may prevent the development of persistent atrophic changes that occur after THR and there is a suggestion by Rasch et al. [11] that fatty infiltration may be reversed by intensive rehabilitation [11].

There have been considerable technical efforts towards optimising surgical treatment of patients with arthritis of the hip, for example with over 100 varieties of hip prostheses being available, multiple types of bearing couples and several surgical approaches. As technology and surgical techniques for total hip replacement (THR) improve, patient expectations, including for an early return to normal physical function and activities, have also increased [15]. However, the actual health gain for many of these innovations relative to "standard" THR is small in terms of patient function and quality of life [16].

In the past, a prolonged hospital stay after THR surgery incorporated a period of supervised rehabilitation to try to achieve restoration of physical function. However, due to the introduction of initiatives such as integrated care pathways and considerations of cost and increasing patient satisfaction, the length of hospital stay following joint replacement has been substantially reduced [17]. Mean length of stay after THR over the past decade has declined from 3 weeks to 4 days [18]. Rehabilitation is therefore increasingly important following total hip replacement. The aim of this review is to systematically investigate the literature with regards to the highest-level evidence (randomised controlled trials) for studies of rehabilitation programmes that have tried to improve function after this common surgical intervention.

## Methods

Studies were eligible for the review if they met the following criteria: 1) randomised controlled trial of exercise rehabilitation interventions to improve functional outcome in the post-operative period; 2) target population includes patients undergoing total hip arthroplasty for osteoarthritis; and 3) publication in English language. For the purpose of this review, early interventions occurred ≤1 month after surgery and late interventions were ≥ 1 month after surgery. This distinction is important as it has been noted that muscle strength declines 4% per day during the first week of immobilisation after major surgery, making it important that rehabilitation is commenced as soon as possible afterwards [19].

Studies were identified from computerized search of MEDLINE (1950 to date), EMBASE (1980 to date), and CINAHL databases. A set was created using the terms: 'total hip arthroplasty AND replacement' and yielded 6559 articles. A search strategy was then built by adding the terms 'exercise', 'rehabilitation', 'physiotherapy', 'functional outcome' to 'total hip arthroplasty AND replacement'. Restriction of the articles obtained from the computerised search to randomised controlled trials and ensuring that the intervention was timed in the post-operative period led to the identification of 15 appropriate studies (Table 1). The studies identified were assessed using the following parameters: 1) whether they were home or centre based, 2) the follow up period used for functional assessment, 3) the interval from surgery to the rehabilitation intervention, 4) the exercise intervention carried out, 5) the outcome measures utilised, and 6) any evidence of dislocation as a complication. For the last parameter listed (dislocation), contact by email was made with the author of any study in which the rate of dislocation was not documented in the article.

**Table 1:** Characteristics of randomised controlled trials on hip arthroplasty rehabilitation interventions to improve functional outcome

Article	Number of participants	Site	Follow up period	Interval from surgery to intervention	Exercise intervention	Outcome measures	Dislocation rate	Limitations
Galea et al., 2008 [20]	Home based group (n = 12); Centre based group (n = 11)	Centre and Home	8 weeks	Immediate post-operative period	All participants: Standard inpatient physiotherapy with functional tasks, instructions to take home and 4 visits at home by physiotherapist Home group: Exercise as above with no advice or further instruction Centre group: 2 visits/week for 45 minutes each time. 5 bouts of exercise per week	No differences in both groups at final follow up although all parameters improved significantly from baseline in both groups. Timed up and go: centre 11.1 ± 2.5 s vs home 11.7 ± 1.5. 6 minute walking test: centre 427 ± 78.2 m vs home 457.8 ± 112.2 m. Stair Climb: centre 3.1 ± 1.8 s vs. 2.9 ± 0.5 s.	None recorded	Patients had significant access to advice and physiotherapy visits. Even though they had the instructions and no advice in the home group, as part of standard protocol, they could all see physiotherapists on a further 3 or 4 occasions if they requested it.

Giaquinto et al., 2010 [21]	Control (n = 33), Intervention (n = 31)	Centre	6 months	< 10 days	Control group: Physiotherapy + 'neutral' massage of scar Intervention group: Hydrotherapy in special pool for 40 minutes after 20 minutes of passive joint exercises All sessions performed x6/week for 3 weeks	At 6 months: WOMAC pain subscale: No pain 45.6% intervention group vs. 23% control WOMAC stiffness subscale: No stiffness 67.7% intervention group vs. 35.8% control WOMAC function subscale: Score of 0 in function 19.3% intervention group vs. 2.56% control	None recorded	-3 week follow-up data initially reported by authors showed objective improvements in speed; stance for example but no further attempt was made to see if this was maintained at 6 months. -No absolute values of the WOMAC subscales given? effect sizes between groups

Greameaux et al., 2008 [22]	Intervention n = 16; Control n = 16	Centre	45 days	Immediate post-operative period	Intervention group: low frequency electrical stimulation of both quadriceps and calf muscles bilaterally. 1 hour session 5 days a week for 5 weeks and conventional physiotherapy (2 hours a day for 5 days/week for 5 weeks (25 sessions)) Control group: Conventional physiotherapy - range of motion exercises, muscle strengthening static and dynamic	Maximal isometric knee extension: Significant ↑ in power of operated limb for intervention vs control (66.7 N(77%) vs 26.7 N(23%)), *p *< 0.05. No significant difference for length of stay nor walking assessment (6MWT and 200 mFWT)	None recorded	-Small sample size -Absence of a true placebo group -Absence of standardisation for the rehabilitation programme

**Article**	**Number of participants**	**Site**	**Follow up period**	**Interval from surgery to intervention**	**Exercise intervention**	**Outcome measures**	**Dislocation** **rate**	**Limitations**

Hesse et al., 2003 [23]	Control n = 40 Intervention n = 40	Centre	12 months	Within 3 weeks post-operatively	All patients: 45 minute individualised treatment on each of 10 consecutive days including passive physiotherapy (massage, heat ultrasound), group therapy in pool. Control: Passive hip and knee mobilisation, strengthening of hip abductor and extensor muscles, gait retraining on floor and stairs Intervention: Treadmill training after above hip and knee mobilisation(20 min days 1-5); days 6-10, 35 minutes treadmill training with 10 minutes physiotherapy	Primary outcome: Harris Hip Score: Intervention vs. control significantly higher (*p *< 0.0001) at 10 days (13.6 points), 3 months (8.9 points) and 12 months (16.5 points). Secondary outcomes: No change in walking velocity between groups Mean interval to abandon crutches 3.2 wks intervention vs 7.9 wks control At end of 10/7 program, for intervention group: Hip extension deficit 6.8° less Gait symmetry 10% greater Affected hip abductor stronger Amplitude of gluteus medius activity 41.5% greater (ALL above *p *< 0.0001) Above differences persisted at 3 and 12 months	None recorded	37.5% drop out rate at 1 year

Husby et al., 2009 [24]	Control (n = 12) Intervention (n = 12)	Centre	5 weeks	Within 1st week postoperatively	Control: Inpatient rehabilitation treatment with sling exercise therapy in hip abduction/adduction, flexion/extension; low resistance exercises for 1 hour, 5 days a week for 4 weeks. Patients discharged before 4 weeks had outpatient treatment 3×/week and were encouraged to do exercises at home 2×/week. Intervention: Above regime and 5 training bouts per week: ~10 minute warm up then stationary cycling at Vo2 max 50%; strength training with 2 exercises leg press and hip abduction on operated leg only. 4 series with rest periods of 2 minutes	Bilateral leg press: 40.9% improvement in intervention vs. control group at 5 weeks (*p *< 0.002). Operated leg strength increased by 65.2% vs. control at 5 weeks (*p *< 0.002) Abductor strength in operated leg 87% more pronounced in intervention vs control at 5 weeks (*p *< 0.002). No difference in gait parameters and health related quality of life outcomes (SF36) at 5 weeks between groups. For work efficiency, the intervention lowered the heart rate by 11.4% relative to the control group at 5 weeks and it also led to a 32.3% trends towards better work efficiency (*p *= 0.065) after 5 weeks.	None recorded	Lack of adequate sample size to demonstrate significant differences in parameters used to assess work efficiency

**Article**	**Number of participants**	**Site**	**Follow up period**	**Interval from surgery to intervention**	**Exercise intervention**	**Outcome measures**	**Dislocation** **rate**	**Limitations**

Jan et al., 2004 [25]	Control (n = 29), Intervention (n = 29	Home	12 weeks	At least 1.5 years	Control group: no exercises Intervention group: 12 week exercise program inclusive of hip flexion range of motion, isotonic strengthening of hip flexors, extensors and abductors using ankle weights, walking + weekly telephone calls	Strength measured with an isokinetic dynamometer. High compliance intervention group, n = 13(> 50% adherence to protocol), showed significant improvement in strength of hip abductors, flexors and extensors on both operated and non-operated legs, as well as greater walking speed and functional activity component of Harris hip score compared to low compliance group, n = 12 and normal control.	None recorded	Subjects in the intervention group were not allowed to visit any physiotherapy department but if they raised issues with the program, they were invited to return to the laboratory for further instructions. No detail is given as to what proportion of the cohort required this and how often.

Jesudason et al., 2002 [26]	Intervention n = 21; Control n = 21	Centre	7 days	1st post-operative day	Control group: Standard protocol for mobilisation, progression of mobility as determined by treating physiotherapist Exercise group: Bed exercises; hip, knee, ankle range of movement exercises. Progressed from 5 repetitions once a day to 10 repetitions as tolerated 2-3 times per day	Pain severity: Significant ↓ in pain (*p *= 0.01) in both groups from days 3-7 post-op. No significant difference in both groups in terms of hip flexion, abduction range of movement, function using the ILOA scale, or length of stay at 3 or 7 days post-operatively.	None recorded	Short intervention Short period of follow up No objective assessment of muscle strength

Liebs et al., 2010 [27]	Hip arthroplasty subgroup. Control n = 104; Intervention n = 99	Centre	24 months	2 weeks postoperatively	Control: No ergometer cycling Intervention: Physiotherapist guided sessions with ergometer initially. Sessions 3/week for ≥3 weeks. All patients: standard program of physiotherapy including range of motion exercises, ADL based movements and walking on stairs and uneven surfaces	Primary outcomes: WOMAC function subscale: Intervention more improved than control at 3 months (16.4 vs. 21.6 (*p *= 0.046)) and 24 months (9 vs. 14.7 (*p *= 0.019)) Secondary outcomes WOMAC stiffness subscale: Intervention more improved than control at 24 months (13.4 vs. 18.6 (*p *= 0.047)) WOMAC pain: Intervention more improved than control at 3 months (11.1 vs. 15.9 (*p *= 0.049)) Improvements also noted in intervention vs control in Lequesne hip and knee score (at 24 months), SF36 (6 and 24 months) and patient satisfaction (92% vs. 80%)	1 dislocation in both groups	Mixed hip and knee arthroplasty population 77% follow up at 24 months

**Article**	**Number of participants**	**Site**	**Follow up period**	**Interval from surgery to intervention**	**Exercise intervention**	**Outcome measures**	**Dislocation** **rate**	**Limitations**

Mahomed et al., 2008 [28]	Home based n = 115; Centre based n = 119	Home/Centre	12 months	On discharge from hospital	All patients: standard physiotherapy regimen: deep breathing, coughing, active and assisted bed/chair gait training Home regime: Referral to community team: nursing, home support etc. Patients discharged when functionally improved as determined by attending therapist Centre-regime: 14 day stay in rehab centre with established pathway (regime not specified)	Primary outcomes: WOMAC function subscale: no difference between groups at 3 and 12 months Hip and Knee satisfaction scale: no difference between groups at 3 and 12 months SF36 short form: no difference at 3 and 12 months Impatient rehabilitation more expensive than home based ($14531 vs. $11082)	2% dislocation rate in both intervention and control groups	Hip and knee arthroplasty patients included. No specific detail given for hip population

Munin et al., 1998 [29]	Mixed hip and knee arthroplasty. Total n = 70 Hip cohort: Control n = 12; Intervention n = 14	Centre	16 weeks	Immediate post-operative period	Intervention group: Commenced rehabilitation protocol at 3 days post-op Control group: Commenced rehabilitation protocol at 7 days post-op.	Median length of stay: intervention 12.2 days vs. control 14.8 days Cost of surgery and rehabilitation lower for intervention ($28256) than control ($29437). RAND 36 functional self assessment: No difference between both groups through the follow-up period	1 dislocation each in control and intervention groups	Mixed hip and knee arthroplasty population Analysing both hips and knees together, the intervention group shows more rapid attainment of short term functional milestones such as ambulation, walking distance and stair climbing ability at 6-10 days post-op. No difference existed for stratifying patients to type of surgery.

**Article**	**Number of participants**	**Site**	**Follow up period**	**Interval from surgery to intervention**	**Exercise intervention**	**Outcome measures**	**Dislocation** **rate**	**Limitations**

Rahmann et al., 2009 [30]	Control n = 17, Aquatic group n = 18, Water exercises n = 19	Centre	180 days	From post-op days 4 - 10	All patients: Standard physiotherapy x1/day Ward control: as above Water exercise group: General exercises in water but not targeted at specific functional retraining in the aquatic environment (40 minutes once daily till discharge) Aquatic group: Hip abductor/adductor exercises with increasing progression- squat, heel raises in various positions in pool (40 minutes once daily till discharge)	Hip subgroup: No significant difference across the 3 groups for primary outcomes such as hip abductor strength, 10 m walk, WOMAC score and secondary outcomes such as timed up and go, quadriceps strength.	None recorded	Mixed group of hip and knee arthroplasty patients Small number of participants

Smith et al., 2008 [31]	Control n = 30; Intervention n = 30	Centre	6 weeks	Immediate post-operative period	Control group: Standard gait re-education protocol from post-operative day 1 Intervention group: Gait re-education with programme of bed exercises from day 1 including; active hip flexion, ankle dorsi/plantarflexion, static quads and gluteal exercises. 10 repetitions each, 5 times a day during hospital stay. Patients encouraged to continue same regime on discharge	Iowa level of assistance (ILOA): Significant improvement from baseline in both groups but no difference in both groups at 3 days and 6 weeks SF12: No difference in both groups	At week 6, 1 dislocation in control group; no dislocations recorded in intervention group	No concealed allocation of randomisation so possible selection bias No objective assessment of hip strength performed

Stockton et al., 2009 [32]	Control n = 27; Intervention n = 30	Centre	6 days	Immediate post-operative period	Control group: Once daily physiotherapy including mobilisation exercises and transfer practice. Encouragement to perform 4× daily till independently mobile Intervention group: 2 physiotherapy sessions per day. Similar protocol to above	Length of stay: No significant difference -Intervention (8.2 days) vs control (8 days) ILOA: Significant difference at 3 days (intervention 28.5 vs control 32.2 (*p *= 0.041) but not at 7 days (intervention 18.2 vs control 20.6)	None recorded	Length of follow up No objective measurement of muscle strength

**Article**	**Number of participants**	**Site**	**Follow up period**	**Interval from surgery to intervention**	**Exercise intervention**	**Outcome measures**	**Dislocation** **rate**	**Limitations**

Suetta et al., 2004 [14]	Total n = 36; Standard rehabilitation (SR) n = 12, Electrical stimulation (ES) n = 11, Resistance training (RT) n = 13	Centre/Home	12 weeks	Immediate post-operative period	SR: 15 exercises in 2 parts. 1^st ^part 6 bed exercises; 2^nd ^part knee extensions in seated position and hip abduction, knee flexion, step training and calf stretching while standing. The attending physiotherapist added ambulation and transfer during the inpatient stay. Exercise was encouraged in the home setting 2×/day and attendance was arranged at a physiotherapy department once a week. NM: Electrodes placed over quadriceps of operated leg 5 cm below inguinal ligament and 5 cm above patella. Pulse rate 40 Hz, pulse width 250 μs, stimulation ~10 s with 20 s of rest. Total stimulation 1 hour per day for 12 weeks. RT: Unilateral progressive resistance training for quadriceps of operated leg. Exercises included knee extension in seated position with sandbags on ankles, leg presses in supine position, supervised by trained physiotherapist. Intensity increased from 50% of 1RM in week 1 to 65% 1RM weeks 2-4, 70% 1RM weeks 5, 6 and 80% 1RM last 6 weeks. For each session patients performed 3-5 sets of 10 repetitions during weeks 1-5 and 2-5 sets of 8 repetitions weeks 6-12.	Length of Stay: RT led to the shortest length of stay compared to ES and SR (10 ± 2.4 days vs. 12 ± 2.8 and 16 ± 7.2 respectively). The difference (37%) between RT and SR was statistically significant (*p *< 0.05). Functional performance: Gait speed: RT ↑ maximal gait speed by 30% at 12 weeks (*p *< 0.001) whilst ES increases it by 19% (*p *< 0.05). No increase was seen in the SR group. Sit to stand: RT ↑ 30%, ES ↑ 21% (both *p *< 0.001) at 12 weeks. SR no improvement. Stair Climb: RT↑ 28% (*p *< 0.005), ES 21% (*p *< 0.001). SR no improvement. Quadriceps cross sectional area (CSA): At 12 weeks, CSA of operated leg was ↑12% in RT group, ↑7% in ES group and ↓9% in SR group (all *p *< 0.05). The non-operated leg was unaffected in all the groups. Peak torque on operated leg at 12 weeks was ↑22% in RT group (*p *< 0.05) and unchanged in ES and SR groups. No change was noted in any of the groups for the non-operated leg.	None recorded	No assessment of compliance in the SR group No documentation as to whether ES group received additional support for ambulation and transfer No use of subjective outcome measures Length of stay assessed was cumulative: did not discriminate between acute surgical inpatient stay and rehabilitation centre length of stay

**Article**	**Number of participants**	**Site**	**Follow up period**	**Interval from surgery to intervention**	**Exercise intervention**	**Outcome measures**	**Dislocation** **rate**	**Limitations**

Trudelle-Jackson et al. 2004 [5]	Control n = 14; Intervention n = 14	Home	8 weeks	4-12 months post-operatively	Control: 7 basic isometric and active range of movement exercises including the glutei, quads, hamstring sets, ankle pumps, heel slides, hip abduction in supine position and hip internal and external rotation in supine position. Intervention: Sit to stand, unilateral heel raises, partial knee bends, 1-legged standing stance, knee raises with alternate arm raise, side and back leg raises in standing, unilateral pelvic lowering and raising in standing Both groups: Progressively increasing repetitions of exercises encouraged 3-4/week for 8 weeks	No difference in fear of falling between both groups. Significant increase in following in the intervention group compared to control at 8 weeks: Hip flexor strength (↑47.8%) Hip extensor strength (↑41.2%) Hip abductor strength (↑23.4%) Postural stability (↑36.8%)	None recorded	Not clear whether the intervention and control groups both received the same amount of encouragement with the visits to increase repetitions Short follow up period

## Results

From Table 1, it can be seen that 11 interventions were performed in a rehabilitation centre, 2 involved a direct comparison between home and centre based interventions and 2 trials were home based. The data shows that, if early intervention is defined as commencing within a month of surgery, such an intervention is more likely to have a beneficial effect if it is performed in a centre and involves progressive resistance training (PRT; i.e. strength training wherein the resistance (weight) lifted is increased in accordance with improved strength to ensure maintenance of a constant relative intensity) (Table 2; 6 out of 6 centre based studies [14,21,23,24,26,27] involving resistance training proved beneficial). The only centre based intervention, by Gremeaux et al., [22], that led to significant improvements in muscle strength without progressive resistance training utilised electrical stimulation. This has been shown by Suetta et al. [14] to not be as efficacious when directly compared to PRT (Table 1). Both home based intervention studies identified in this review [5,25] were carried out in the late phase (> 1 month post-operatively) and led to significant improvements in functional outcome parameters after short periods of follow up (8 and 12 weeks respectively). The other 6 studies reviewed include 2 comparing home and centre based interventions [20,28] and 4 others performed in the early phase in a centre setting [29-32] but without the use of progressive training. Their limitations and findings are as detailed in Table 1.

**Table 2:** Timing and effects of rehabilitation interventions following hip arthroplasty

Article	Intervention site	Timing of intervention: Early (> 1 month) or late (> 1 month)	Use of progressive resistance training? Yes/No	Significant effect of intervention on functional outcomes measured? Yes/No
Giaquinto et al., 2008 [21]	Centre	Early	Yes	Yes

Husby et al., 2009 [24]	Centre	Early	Yes	Yes

Galea et al., 2008 [20]	Home/Centre	Early	No	No

Smith et al., 2008 [31]	Centre	Early	No	No

Rahmann et al., 2009 [30]	Centre	Early	No	No

Liebs et al., 2010 [27]	Centre	Early	Yes	Yes

Mahomed et al., 2008 [28]	Home/Centre	Early	No	No

Hesse et al., 2003 [23]	Centre	Early	Yes	Yes

Munin et al., 1998 [29]	Centre	Early	No	No

Gremeaux et al., 2008 [22]	Centre	Early	No	Yes

Jesudason et al., 2002 [26]	Centre	Early	Yes	Yes

Suetta et al., 2004 [14]	Centre	Early	Yes	Yes

Trudelle-Jackson et al., 2004 [5]	Home	Late	Yes	Yes

Jan et al., 2004 [25]	Home	Late	Yes	Yes

Stockton et al., 2009 [32]	Centre	Early	No	No

The follow up periods for the centre based studies varied from 7 days to 24 months (Table 1). In terms of the follow up periods that were longer than the interventions used, Liebs et al. [27] show with their ergometer study that the benefits of a resistance program are sustained for 24 months from surgery which helps to address the functional deficits identified after THR. Mahomed et al. [28] demonstrated that there is not much difference at 1 year between home and centre based post-THR standard physiotherapy interventions in terms of subjective functional outcome (measured with the WOMAC), but there was no progressive training included in the prescribed programs used and this may explain the lack of a significant difference between the groups.

## Discussion

'Standard physiotherapy', (i.e. not involving resistance training) following major surgery enables most patients to regain basal levels of function but leaves them with significant muscle wasting as it lacks the intensity of exercise required to elicit muscle hypertrophy [14,33]. The most commonly used rehabilitation regimes for elderly individuals are based on functional types of exercises without external loading. However, this type of intervention not only fails to elicit increases in muscle mass but does not prevent further muscle atrophy [34]. In contrast, high-intensity PRT is an extremely effective and safe method for inducing muscle hypertrophy and increasing muscle strength and subsequently improving functional performance in healthy individuals, those with chronic disease e.g. rheumatoid arthritis [33], and the elderly [35-37]. An unpublished survey from our institution of physiotherapy practice around the UK after THR revealed that 73% of qualified physiotherapists knew what progressive resistance training entailed but only 32% used it in their prescribed programmes after THR.

PRT typically elicits positive health and performance adaptations by challenging the skeletal muscles with loads that can be lifted repetitively for 8-15 repetitions maximum (RM) per set before the onset of neuromuscular fatigue i.e. the point at which appropriate technique can no longer be maintained [3,38]. PRT sessions are optimal when followed by periods of recovery ranging from 48 to 72 h to allow for physiological super compensation (i.e. positive adaptation)[39]. To facilitate continued adaptation and avoid the onset of a plateau in physiological adaptation, training intensity (i.e. load) and/or training volume (i.e. the total number of lifts) are progressively increased in line with training response [39]. Health and performance are maintained with continued training [40] and PRT is well established as a safe and beneficial exercise modality for individuals of all ages and fitness levels, including those afflicted with severe chronic illnesses [33,39,41]. Additionally, PRT is particularly beneficial for elderly individuals given its efficacy in counteracting sarcopenia, reducing fat mass, abating osteoporosis, and reversing the many physiological and functional impairments that accrue with age [4-6]. The positive benefits of this method of rehabilitation are evident with the 5 randomised controlled trials identified in this review (Table 2).

A major disadvantage of programs used in the post-operative period following THR is the need for patients to exercise under the supervision of professional staff at a hospital or rehabilitation centre [20]. This makes program delivery expensive due to the high costs associated with supervision and transport. In addition, some THR patients are excluded because of difficulties with mobility [7]. A similar exercise regime that could be performed at home would overcome these cost and logistic implications.

A limitation of the home-based interventions is that follow-up did not extend beyond the end of the exercise interventions periods. Thus, it is not clear whether the benefits evident at the end of the exercise intervention are maintained in the longer term. The other obvious shortcoming is the lateness of the intervention in the home setting and consequently the failure to ameliorate or prevent the exacerbated loss of muscle and function after surgery. A recent systematic review by Di Monaco et al. [42] suggests that the difficulties in THR rehabilitation research are that there is a lack of multicentre clinical trials with large sample sizes to inform the design of optimal physical exercise programs.

It follows that with the adverse impact of major surgery on muscle mass and therefore strength and function, being able to provide an intervention in the early post-operative period is essential. The intervention should also obviate the problems of cost and transport that supervised, centre-based rehabilitation programs necessarily involve. Providing patients with the impetus to rehabilitate themselves with minimal supervision in their home environments may be the answer.

A major concern with orthopaedic surgeons is dislocation of the hip arthroplasty (incidence after primary THR of 1-9.5% [43] on patient mobilisation and the instructions patients have to adhere to afterwards take this into account. These include a restriction of hip flexion to less than 90°, no crossing of the legs, and elevation of toilet seats and chairs in the house etc. From this systematic review of 15 randomised controlled trials, the dislocation rate in the pooled sample of patients who underwent the interventions described was 0.77% (4 recorded dislocations in a pooled sample of n = 516) whilst the rate in patients who were in the normal control groups was 1% (5 recorded dislocations in a pooled sample of n = 505). Thus, it is safe to conclude that these exercise programmes are not associated with an increased risk of dislocation.

## Conclusions

Total hip replacement surgery provides good relief for patients' pain but fails to fully restore physical function. This systematic review demonstrates that significant improvements in muscle strength and function are achievable with PRT. Regardless of the timing of the intervention, PRT appears to have a significant benefit on patient function following THR. Late PRT interventions do work and are safe, and they have been performed mainly in the home setting but the studies done have short periods of follow up and have a further limitation of the pre-existing functional deficit due to the timing post-operatively. Early PRT regimes identified in the studies reviewed in this article have shown the need for a centre-based approach and this has demonstrable benefit but there are issues of high costs of transport and supervision. Early home based PRT studies that are effective and safe; with adequate follow-up after THR surgery would potentially address these issues.

## Competing interests

There are no competing interests to declare in the preparation of this manuscript.

## Authors' contributions

TO carried out the review of the literature, wrote and made changes to the final version of the manuscript as advised by AL, PM and JA (Academic supervisors). All authors read and approved the final manuscript

## Funding

This work was supported by a grant from the Betsi Cadwaladr University Health Board Small Grants Committee.

## Ethical standards

There were no ethical considerations in the preparation of this review
